# Who Is a Doctor?

**Published:** 1861-07

**Authors:** 


### Art. V.—WHO IS A DOCTOR?

The equanimity of the profession has of late been somewhat ruffled from time to time by the agitation of a question which, to the uninitiated, might seem to imply that the dignity of medicine, as represented by orthodox practitioners of physic, was, in some inexplicable manner, at stake. The differential characters of a Doctor constitute the subject-matter of the disputation—*Who is a Doctor?*—Who is *not* a Doctor? The late M. Ferrus, on one occasion being a guest along with sundry literary and scientific celebrities at the house of Alibert, and the conversation having fallen upon the trades and professions of France, asserted that of all professions medicine was undoubtedly that which was most followed, not only in Paris, but in all countries. Every one dissented, and Alibert foremost of all. "Why," said he, "there are not a thousand doctors in Paris, and some villages in the provinces have no doctors at all." Ferrus, apparently silenced by the overwhelming majority against him, ended by saying that he was sure that the company would ere long find that he was right. In the course of the evening, throwing himself into an arm-chair, and burying his face in a handkerchief, he began to groan most piteously. In an instant he was surrounded by the whole party, who sympathizingly inquired the cause of his distress. "A violent toothache," was the answer. "Go home, and gargle with warm milk," said one; "Put a little cotton-wool steeped in laudanum into your ear," said another; a third recommended a poultice of boiled figs; a fourth a stocking-full of hot sand; a fifth (and this no less a person than Humboldt) the repetition of some charming couplet which Brazier had just sung; and so on until each of the company had recommended some infallible recipe. When the last of the panaceas was exhausted, Ferrus, throwing away his handkerchief, and desisting from his grimaces of feigned agony, burst into a hearty laugh, saying, "Well, was I not right?

You are all doctors, and have each furnished me with a prescription. In France no one believes in medicine, and yet each one is a physician. Will you still venture to deny that my assertion was correct?"*

The true question at issue, however, in the present discussion is not who is duly authorized to practise the art and science of medicine upon Her Majesty's lieges, but who is entitled to use the style and title of Doctor of Physic?—and still more explicitly whether the licence of a College of Physicians, or degree of Bachelor of Medicine, gives the holder rightfully the privilege of designating himself Doctor, and attaching to his name the honourable letters M.D.?† But for the fact that the question has arisen, it is almost inconceivable that it should have arisen, since it is not easy to imagine that the relationship between a simple licence to practise, and a scholastic degree of the highest grade, is so close, that the former could be regarded as equivalent to the latter,‡ or that the lowest degree of a faculty should confer the right of assuming the highest. But, be this as it may, it is undoubtedly the common usage both for Physicians who are simply such, and for Bachelors of Medicine, not only to style themselves, but to be styled Doctors, as if Doctors of Medicine.

Mere convenience, setting courtesy aside, would, it may readily be believed, lead to the appellation of Doctor being given to the Bachelor of Medicine practising as a physician, and to his adopting the title of the highest degree of his Faculty in ordinary life. Beyond the precincts of his University, the lower and less familiarly-named degree would have no meaning, while practically it yielded all the advantages of the higher and more familiarly named. Again, until a comparatively recent period, from medical instruction being confined to Universities, the orthodox Physician was almost of necessity a graduate in medicine. Hence, in the eyes of the people, the Physician became, and was for ages identified with, the Doctor of Medicine. The Physician, by virtue of a simple licence to practise as such, only dates from that by no means remote period of time when schools of physic sprang up apart from Universities. But, since in his professional relations with the world at large the Physician *per se* differs in no

---

* We are indebted to the Paris correspondent of the *Lancet* (April 6, 1861) for this interesting anecdote of M. Ferrus.

† The new order of Licentiates of the College of Physicians of London are expressly debarred from adopting the title of Doctor, and we believe also of Physician, upon the strength of the licence alone. The present *Member* of the College is the equivalent of the *Licentiate* prior to the adoption of the recent regulations affecting general practitioners. The Member is, indeed, the Physician proper of the present arrangements.

‡ The King's and Queen's College of Physicians of Ireland claims for its *licentiates*, by charter, the right to the title of Doctor of Medicine.

respect from the Doctor of Medicine practising as a physician, public faith in the belief that the terms Physician and Doctor of Physic are of the self-same value has not been shaken, and convenience and courtesy have helped to maintain the custom of addressing the Physician, under all circumstances, by the style and title of Doctor.

Common usage, however, forms but an unstable foundation upon which to build a rightful claim to a title which especially appertains to, and is the distinctive mark of, a University degree. It is argued, therefore, in addition, that the Physician, in so far at least as his medical training is concerned, stands fully on a par with the Doctor of Medicine, and indeed, that his licence is in many instances a diploma of greater merit than the degree of several Universities. This may suffice to show the worthiness of the Physician's licence, but not to prove that it justifies the possessor in asserting the right to add on that account the term Doctor to his name—for Doctor is properly no addition but a degree (*quia gradatim et progressione Doctrinæ provenit*). And admitting even that the degree has been prostituted in several Universities, this surely can be no reason for divesting it to a still greater extent of its legitimate signification.

It is by no means a new thing to attempt to elevate the status of the Physician at the expense of the Degree in Medicine, although it is a somewhat paradoxical method of proceeding, by which, as in the present case, additional dignity is sought by dragging what we aim at down to our own level. In the earlier contentions of the College of Physicians with graduates of the Universities, the College, notwithstanding that it was then comparatively a recent creation, boldly sought to place itself on an equality with, if not to take the wall of, the Universities. But it is not to be forgotten that in that day the licentiates of the College were without exception graduates, and it is to be presumed they conceived that their licence added fresh lustre to their degree. Lord Chief Justice Coke, however, observed, in the celebrated case of Dr. Bonham, that "Because that much was said in commendations of the Doctors of Physick of the said College within London" [not, be it remarked, meaning to imply that they were Doctors of Physic because members of that College, but simply speaking of them according to the degree which as previously observed they possessed], "and somewhat as he conceived in derogation of the dignity of the Doctors of Universities, he first attributed much to the Doctors of the said College within London, and did confess that nothing was spoken which was not due to their merits; but yet that no comparison was to be made between that private College and any of the Universities of Cambridge and Oxford, no more than between the Father and his Children, or between the Fountain and the small Rivers which descend from thence. The University is *Alma Mater*, from whose breasts those of that private College have sucked all their science and knowledge (which I acknowledge to be great and profound), but the Law saith, *Erubescit lex Filios castigare Parentes.* The University is the fountain, and that and the like private Colleges are *tanquam rivuli*, which flow from the fountain, *et melius est petere fontes quam sectari rivulos.* Briefly, *Academiæ Cantabridgiæ et Oxoniæ sunt Athenæ nostræ nobilissimæ, regni soles, oculi et animæ regni, unde Religio, humanitas et doctrina in omnes regni partes uberrimæ diffunduntur.* But it is true, *Nunquam sufficiet copia laudatoris, quia nunquam deficiet materia laudis;* and therefore these Universities exceed and excel all private Colleges, *tanquam inter viburna cupressus.* And it was observed that King Henry VIII. his said Letters Patent, and the King and Parliament in the Act of 14 H. VIII., in making of a Law concerning Physicians, for the more safety and health of men, therein have followed the order of a good Physician (*Rex enim omnes artes censetur habere in scrinio pectoris sui*). For, *Medicina est duplex, removens et promovens; removens morbum, et promovens ad salutem.* And therefore five manner of persons (who more hurt the body of men than the disease itself) are to be removed. 1. *Improbi;* 2. *Avari, qui medicinam magis avaritiæ suæ causâ quam ullius bonæ conscientiæ fiducia profitentur;* 3. *Malitiosi;* 4. *Temerarii;* 5. *Inscii;* and of the other part, five manner of persons were to be promoted, as appeareth by the said Act, *scil.*, those that were, 1. profound; 2. sad; 3. discreet; 4. groundedly learned; 5. profoundly studied. And it was well ordained, that the professors of Physick should be profound, sad, discreet, &c., and not youths who have no gravity and experience; for as one saith, *In juvene Theologo conscientiæ detrimentum, in juvene Legista bursæ decrementum, in juvene Medico cœmeterii incrementum.* And it ought to be presumed every Doctor of any of the Universities to be within the Statute, *i.e.*, to be profound, sad, discreet, groundedly learned, and profoundly studied, for none can there be Master of Arts (who is a Doctor of Philosophy) under the study of seven years, and cannot be Doctor of Physick under seven years more in the study of Physic."

The Doctor of Medicine, indeed, is, by virtue of his degree, necessarily a physician, but the physician is not, by virtue of his licence, necessarily a Doctor of Medicine. Letting, therefore, the common usage of addressing the physician as "Doctor" stand for what it is worth in courtesy or convenience, we dissent from the proposition that the physician's licence entitles the holder to the same honorary appellation as the Doctor's degree.

It is curious that this question should have arisen at a time when the degree or title of Doctor is no longer the distinctive mark of the Physician, but is common also to the Surgeon and General Practitioner. That exclusiveness which so long, and so injuriously to the best interests of physic, restricted the practical utility of a Doctor of Medicine's degree to a physician's duties, is passing away; and the degree is being rapidly restored to its true position and dignity as the culminating distinction of medicine in its entirety, and not as the symbol of one branch of medicine only. The degree, in fact, is becoming divested of the class character which heretofore was so strongly attached to it; and it is now rightfully borne alike by the general practitioner, the surgeon, and the apothecary. Unhappily, this change of feeling, in the beginning, was turned to profitable account by sundry Universities, and facilities were offered by them for obtaining degrees, which threatened in the end to make the title of "Doctor" a term of contempt in the eyes of the public. At one time it seemed not improbable that the title might become of as little estimation as with a certain Wensleydale dame, who, on being asked why she persisted in calling a charlatan of local celebrity *Doctor*, instead of *Mister*, he having no right to the medical title, replied, "I'll call him nought else. What mun a body *mister* sic chaps as him for? *Doctor's* good enough for sic-like folk."

The more culpably lax methods of granting degrees are, no doubt, now pretty effectively curbed by recent legislation, and by the growth of a more healthy feeling among the profession as to the true value of a degree. The question of *Who is a Doctor?* shows, however, that even among the ranks of those who have been customarily looked upon as the elect of the profession, there is still something to be learned before the dignity of the medical man is rightly apprehended. For our own part, we do not see the need of any adventitious aid to the time-honoured name of Physician, and we would hold up as an example worthy of honour that gentleman who, possessing solely the Licence of the College of Physicians, is content to describe himself upon his door-plate as, *Mr.* ——, Physician. If, however, the present discussion should be persisted in, we would suggest that an extra-academical claim to the title of Doctor will probably be most surely maintained upon a dictum of the Lord Chief Baron in the case of *Ellis v. Kelly*, to wit, that "If a man be registered, he may call himself what he likes."

